# Feeding in the first six months of life is associated with the probability of having bronchiolitis: a cohort study in Spain

**DOI:** 10.1186/s13006-021-00422-z

**Published:** 2021-10-18

**Authors:** Inés Gómez-Acebo, Carolina Lechosa-Muñiz, María Paz-Zulueta, Trinidad Dierssen Sotos, Jéssica Alonso-Molero, Javier Llorca, María J. Cabero-Perez

**Affiliations:** 1 Universidad de Cantabria – IDIVAL, Santander, Spain; 2 CIBER Epidemiología y Salud Pública (CIBERESP), Madrid, Spain; 3 Hospital Universitario Marqués de Valdecilla, Santander, Spain; 4 Universidad de Cantabria, Santander, Spain

**Keywords:** Breastfeeding, Breastfeeding duration, Feeding type, Feeding trajectory, Bronchiolitis, Spain

## Abstract

**Background:**

Breastfeeding is associated with lower incidence and severity of lower respiratory tract disease. However, little is known about the relationship between feeding type and breastfeeding duration with bronchiolitis in a child’s first year.

**Methods:**

A prospective cohort study of 969 newborn babies were followed-up for 12 months to determine breastfeeding duration, feeding type, feeding trajectory, and bronchiolitis episodes at Marqués de Valdecilla University Hospital, Spain in 2018. Type of feeding was recorded by interviewing mothers at the time of hospital discharge and at 2, 4, 6, 9 and 12 months of life, in three categories: breastfeeding, mixed feeding and infant formula. Type of feeding at hospital discharge refers to feeding from birth to discharge. In any other times studied, it refers to feeding in the last 24 h. The association between the feeding type and bronchiolitis was analysed using logistic regression. Poisson regression was used to evaluate the association between feeding type and the number of bronchiolitis episodes with Kaplan-Meier estimators presenting the cumulative probability of suffering bronchiolitis. The results were adjusted for mother and child characteristics.

**Results:**

Our data shows exclusive breastfeeding and mixed breastfeeding reduce the number of episodes of bronchiolitis. Regarding feeding at 4 months, exclusive breastfeeding reduced by 41% the number of episodes of bronchiolitis (adjusted incidence Ratio (aIR) 0.59, 95% CI 0.46, 0.76) and mixed feeding by 37% (aIR 0.63, 95% CI 0.47, 0.86). Moreover, changing from exclusive breastfeeding to mixed feeding increased the incidence of bronchiolitis compared with continuing exclusive breastfeeding. An early swap to mixed breastfeeding before months 2 or 4, was associated with a reduced the number of episodes of bronchiolitis, (aIR 0.53, 95% CI 0.39, 0.73 if introduction of mixed breastfeeding before month 2, and aIR 0.61, 95% CI 0.45, 0.83 if introduction of mixed breastfeeding before month 4), when compared with infant formula alone.

**Conclusions:**

Any breastfeeding was associated with lower incidence of bronchiolitis and number of episodes of bronchiolitis in the first year of life. Consequently, promoting programmes facilitating exclusive or mixed breastfeeding would be a relevant measure in the prevention of bronchiolitis.

## Background

Acute bronchiolitis is a lower respiratory tract infection which affects children under 2 years of age, although it tends to occur in the first year of life [1]. It is usually a moderate illness, but in some infants, it can be more severe and require hospitalization. Approximately 10–15% of children have acute bronchiolitis during the first year of life [2].

Bronchiolitis is usually caused by respiratory syncytial virus (RSV), and is associated with more than 50% of hospitalization in infants [3]. In 2015, it had been estimated that 33.1 million episodes of RSV infection, resulting in about 3.2 million hospital admissions and 59.600 in-hospital deaths, happened globally, in children younger than 4 years [4]. Furthermore, many observational studies have shown that RSV disease in childhood is associated with increased wheezing or asthma later in childhood [2, 5].

Some widely described factors that increase the risk of suffering from acute bronchiolitis are comorbidities such as prematurity, bronchopulmonary dysplasia, or congenital heart disease [6, 7]. Regarding socio-demographic factors, there is a relationship between the incidence of bronchiolitis and maternal youth [8, 9]. Furthermore, factors related to the mother’s nutritional status and her stress level are involved [10]. In addition, attendance at daycare or living with older siblings are also shown as factors that increase risk [8]. Considering external factors that can be modified in the environment of individuals, exposure to tobacco constitutes a well-established risk factor, both for the susceptibility of suffering an episode of bronchiolitis and for it to be more severe [10, 11]. Breastfeeding is associated with lower incidence and severity of lower respiratory tract disease [12–14] and several studies have confirmed that the longer the duration of breastfeeding the better the clinical outcome of the episode of bronchiolitis [15, 16]. In this way, the World Health Organization, as well as scientific societies in America and Europe, recommends exclusive breastfeeding for the first 6 months of life [17]. However, despite the beneficial effects of breastfeeding and the recommendations of different scientific societies, in European countries exclusive breastfeeding rates reached around 56–98% immediately after birth and drop to only 13–39% at 6 months of life [18].

Our main objective was to determine the relationship between type of feeding and duration of breastfeeding and bronchiolitis in the first year of life.

## Methods

### Design and setting

Prospective cohort study that includes 969 newborns recruited consecutively from January 1, 2018 to August 31, 2018, at the Marqués de Valdecilla University Hospital (HUMV), Santander, Spain. The HUMV is a public hospital, which is implemented in the Baby-Friendly Hospital Initiative (BFHI) [19] and attends around 3000 deliveries a year. Details on design and gathering information have been published elsewhere [20]. This manuscript is a further analysis of that sample after following children for 1 year.

### Data collection

Data on pregnancy, delivery, and characteristics before hospital discharge, including type of feeding at discharge, were obtained from obstetrics records. All neonates were followed-up for 12 months to determine the duration of breastfeeding and the presence of bronchiolitis. Information in the follow-up was obtained from paediatric records and via an interview with the mother at the time of hospital discharge and in each regular consultation with the paediatrician. In this regard, the childcare program of the Regional Service of Health established each child should have a paediatrician review at 2, 4, 6, 9 and 12 months of life.

Apart from the main exposure of type of feeding and the event of bronchiolitis, which are further developed, information was gathered about maternal age, educational level, occupational activity, smoking during pregnancy, duration of pregnancy, type of delivery and whether the pregnancy was single or multiple. Information about neonates included sex and birthweight, as well as conditions present at birth. Newborn attendance to childcare was obtained by interviewing the mother in each check-up at 2, 4, 6, 9 and 12 months of life. Attendance to daycare was obtained by interviewing the mother.

### Type of feeding: data management

**Fig. 1:**
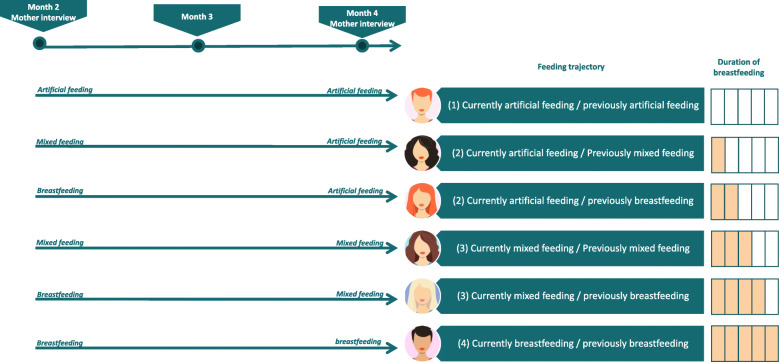
Example of the evolution in the type of breastfeeding in six different women based on the mother’s responses to the two- and four-month interviews

Type of feeding was recorded via interviewing mothers at the time of hospital discharge and at 2, 4, 6, 9 and 12 months of life, in three categories: breastfeeding, mixed feeding and infant formula. In this regard, type of feeding at hospital discharge is considered for infants who have been exclusively breastfed or who have received expressed breast milk from birth to discharge. In the other time points studied the type of feeding refers to the food received in the last 24 h. Breastfeeding was considered that WHO’s definition for “exclusive breastfeeding”, it means that the infant receives since birth breast milk (including expressed breast milk or breast milk from a wet nurse) and allows the infant to receive oral rehydration salts, drops, syrups (vitamins, minerals, medicines), but nothing else (no other food or drink, not even water) [21]. Mixed feeding was when the infant supplemented breastfeeding with infant formula. Infant formula means exclusive formula milk. Then, at each time point (2, 4, 6, 9 and 12 months), we identified the feeding trajectory in four categories: (1) Infant formula only (2) Previously breastfed, currently infant formula only (3) Previously exclusive breastfeeding or any breastfeeding, currently any breastfeeding (4) Exclusive breastfeeding since birth. For instance, a neonate breastfed until month three, then changed to mixed feed until month seven and then changed to formula would be classified as category 4 (breastfeeding) in month 2, category 3 in month 4 as she/he changed from breastfeeding to mixed, category 3 in month 6 as she/he continued with mixed feed and category 2 in month nine. Figure 1 shows an example of the evolution in the type of breastfeeding from 2 months to 4 months in six different women as a function of the mother’s responses to the two- and four-month interviews.

### Bronchiolitis: data management

Diagnosis of bronchiolitis was assessed from paediatric records and interviews with the mother. For this analysis we classified bronchiolitis in three ways: (1) as dichotomic variable (the neonate had bronchiolitis or not), (2) as number of episodes of bronchiolitis in the follow-up, (3) as the period bronchiolitis occurred (cumulative number of episodes of bronchiolitis from birth to 12th month, from 2nd to 12th month, from 4th to 12th month, and from 6th to 12th month). This last classification allowed us to analyse type of feeding at specific time, say 2nd month, bronchiolitis association without risking a reverse causation bias.

### Statistical analysis

Descriptive results are presented as number (percentage) or mean ± standard deviation.

The association between the type of feeding and the presence of bronchiolitis was analysed using logistic regression. The results, adjusted for maternal smoking, maternal occupational status, twin pregnancy, gestation length, birth order, attending daycare and months at which the neonate began kindergarten, are presented as adjusted Odds Ratio (aOR) with 95% confidence intervals (95% CI). The association between type of feeding and the number of episodes of bronchiolitis was assessed by Poisson regression and the results were presented as adjusted incidence rate Ratio (aIR) with 95% confidence intervals.

The cumulative probability of suffering bronchiolitis is presented using Kaplan-Meier estimators. For this analysis, the event was defined as the first diagnosis of bronchiolitis and infants were censored if they did not have bronchiolitis at 12 months of life. A complementary Kaplan-Meier estimator was carried out for repeated events; that is, children were followed for 12 months and each episode of bronchiolitis was considered an event.

All statistical analyses were performed with the Stata 16/SE software (Stata Co., College Station, Tx, US).

### Ethical considerations

This project was approved by the Ethics Committee for Clinical Research of Cantabria on 21 July 2017, reference number 2017.142. During the hospital stay after delivery, mothers were informed of the existence of the study and they were requested to sign the informed consent form to participate in the study. The project was carried out according to the Spanish laws on biomedical research, the European Union regulations on the protection of natural persons with regard to the processing of personal data and the Declaration of Helsinki on ethical principles for medical research involving human subjects.

## Results

The descriptive data of the cohort were previously published [22]. The initial sample was 969 newborns included in the study at birth. Sixty-two children were excluded because it was unknown whether they had bronchiolitis. Finally, 907 newborns out of 888 pregnancies were included in the analysis and 882 children (91%) were followed up to 12 months.

The main characteristics of mothers and children in this cohort are shown in Table 1. Mothers were 33.7 ± 5.2 years old on average and 37% of mothers (*N* = 332) had university studies and approximately 70% worked outside home. Additionally, around 87% of mothers did not smoke during pregnancy. About half of children were females, 5.5% of children were premature, 24% were delivered via Caesarean section and 8% weighed less than 2500 g at birth.

**Table 1:** Main characteristics of participants in the study

		Total	Bronchiolitis				Number of episodes of bronchiolitis		
		***N*** = 907	***N*** = 652	***N*** = 255		***N*** = 652	***N*** = 162	***N*** = 93	
Variable	Category	N (%)	No	Yes	P	0	1	> 1	p
Maternal age (years)	mean ± sd	33.7 ± 5.2	33.84 (0.20)	33.36 (0.32)	0.213	33.84 (0.20)	33.55 (0.41)	33.04 (0.54)	0.348
Maternal educational level	Primary studies	215 (22.7)	144 (72.36)	55 (27.64)	0.643	144 (72.36)	30 (15.08)	25 (12.56)	0.290
	Secondary studies	112 (11.8)	79 (72.48)	30 (27.52)		79 (72.48)	23 (21.10)	7 (6.42)	
	foundation degree	272 (28.7)	180 (68.97)	81 (31.03)		180 (68.97)	48 (18.39)	33 (12.64)	
	University studies	350 (36.9)	249 (73.67)	89 (26.33)		249 (73.67)	61 (18.05)	28 (8.28)	
Maternal occupation	working	660 (69.6)	451 (71.36)	181 (28.64)	0.911	451 (71.36)	121 (19.15)	60 (9.49)	0.655
	unemployed	163 (17.2)	116 (73.89)	41 (26.11)		116 (73.89)	24 (15.29)	17 (10.83)	
	Inactive	116 (12.2)	79 (72.48)	30 (27.52)		79 (72.48)	15 (13.76)	15 (13.76)	
	Student	10 (1.1)	6 (66.67)	3 (33.33)		6 (66.67)	2 (22.22)	1 (11.11)	
Smoking in pregnancy	No	830 (87.5)	571 (72.10)	221 (27.90)	0.711	571 (72.10)	140 (17.68)	81 (10.23)	0.922
	Yes	119 (12.5)	81 (70.43)	34 (29.57)		81 (70.43)	22 (19.13)	12 (10.43)	
Cigarettes/day	mean ± sd		0.12 (0.01)	0.13 (0.02)	0.712	0.12 (0.01)	0.14 (0.03)	0.13 (0.03)	0.923
Pregnancy duration	< 34 weeks	16 (1.7)	10 (50.00)	10 (50.00)	0.038	10 (50.00)	5 (25.00)	5 (25.00)	0.020
	34–36 weeks	36 (3.8)	24 (63.16)	14 (36.84)		24 (63.16)	6 (15.79)	8 (21.05)	
	≥37 weeks	897 (94.5)	618 (72.79)	231 (27.21)		618 (72.79)	151 (17.79)	80 (9.42)	
Type of delivery	Vaginal	653 (67.4)	446 (73.00)	165 (27.00)	0.062	446 (73.00)	105 (17.18)	60 (9.82)	0.216
	Instrumental vaginal	80 (8.3)	60 (78.95)	16 (21.05)		60 (78.95)	11 (14.47)	5 (6.58)	
	Caesarean section	236 (24.4)	146 (66.36)	74 (33.64)		146 (66.36)	46 (20.91)	28 (12.73)	
Newborn gender	Male	490 (50.6)	312 (68.27)	145 (31.73)	0.015	312 (68.27)	83 (18.16)	62 (13.57)	0.003
	female	479 (49.4)	340 (75.56)	110 (24.44)		340 (75.56)	79 (17.56)	31 (6.89)	
Sibling pregnancy	No	929 (95.9)	631 (72.70)	237 (27.30)	0.010	631 (72.70)	155 (17.86)	82 (9.45)	0.001
	Yes	40 (4.1)	21 (53.85)	18 (46.15)		21 (53.85)	7 (17.95)	11 (28.21)	
Newborn weight	< 2500	83 (8.6)	54 (66.67)	27 (33.33)	0.474	54 (66.67)	15 (18.52)	12 (14.81)	0.474
	2500–4000 g	808 (93.4)	545 (72.67)	205 (27.33)		545 (72.67)	130 (17.33)	75 (10.00)	
	> 4000 g	78 (8.1)	53 (69.74)	23 (30.26)		53 (69.74)	17 (22.37)	6 (7.89)	
Attending daycare	No	763 (78.7)	556 (73.84)	197 (26.16)	0.006	556 (73.84)	121 (16.07)	76 (10.09)	0.015
	Yes	132 (13.6)	79 (60.31)	52 (39.69)		79 (60.31)	37 (28.24)	15 (11.45)	
	Unknown	74 (7.6)	17 (73.91)	6 (26.09)		17 (73.91)	4 (17.39)	2 (8.70)	
Breastfeeding duration^a^ (months)	mean ± sd	5.9 ± 5.2	6.22 (0.21)	5.26 (0.33)	0.014	6.22 (0.21)	5.54 (0.41)	4.79 (0.54)	0.026

^a^Any breastfeeding

### Probability of a child suffering bronchiolitis

**Fig. 2:**
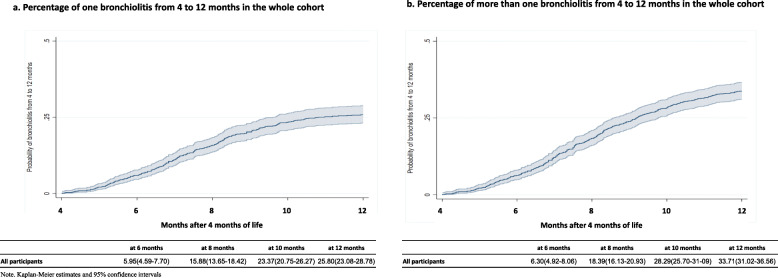
Bronchiolitis in the whole cohort. Kaplan Meier estimates with 95% confidence bands

Over the 12 months, we detected 234 episodes of bronchiolitis in 907 children. About 6% of the children had an episode of bronchiolitis by the 6th month of life and the percentage increased to 16, 23, and 26% at 8, 10, and 12 months after birth, respectively. Figure 2 displays Kaplan-Meier estimates of the probability of suffering at least one bronchiolitis (Fig. 2a) and the cumulative number of episodes of bronchiolitis in the first year of life (Fig. 2b).

Regarding pregnancy duration, 10 out of 20 (50%) were delivered before week 34, 14 out of 24 (37%) whose gestation lasted 34–36, and 231 out of 897 (27%) delivered at term, have had at least an episode of bronchiolitis. Differences were also observed regarding the infant’s sex, 32% of male and 24% of female have had bronchiolitis. When evaluating the number of twins, we observed that 18 children (46%) of twins compared to 27% of singleton have had bronchiolitis. Regarding daycare attendance, 40% (52/132) of infants attending childcare experienced bronchiolitis compared to 26% (197/763) of infants who did not attending it (Table 1). Relationship between feeding and bronchiolitis.

Table 2 shows the association between feeding and risk of developing bronchiolitis in the first year of life. Regarding the type of feeding, breastfeeding at time of hospital discharge was associated with a lowering the risk of developing bronchiolitis by approximately 40% compared to infant formula (aOR 0.61; 95% CI 0.41, 0.91 for exclusive breastfeeding and aOR 0.60, 95% CI 039, 0.94 for mixed feeding). Results at 2nd, 4th and 6th months were similar.

**Table 2:** Relationship between type of feeding and diagnosed with at least one episode of bronchiolitis

	Hospital discharge to 12th month				2nd to 12th month		4th to 12th month			6th to 12th month		
Category	no. bronchiolitis / children at discharge	aOR (95% CI)	p	no. bronchiolitis / children at 2nd month	aOR (95% CI)	p	no. bronchiolitis / children at 4th month	aOR (95% CI)	p	no. bronchiolitis / children at 6th month	aOR (95% CI)	p
**Type of feeding**												
**Infant formula only**	61/163	1(ref.)	.	96/295	1(ref.)	.	118/383	1(ref.)	.	125/477	1(ref.)	.
**Mixed (breastfeeding and infant formula)**	**70/264**	**0.60 (0.39, 0.94)**	**0.02**	44/179	0.69 (0.44, 1.06)	0.09	40/162	0.77 (0.49, 1.19)	0.24	36/181	0.73 (0.47, 1.14)	0.17
**Breastfeeding**	**123/479**	**0.61 (0.41, 0.91)**	**0.02**	105/418	0.73 (0.51, 1.05)	0.09	73/348	0.61 (0.43, 0.89)	**0.01**	42/234	0.67 (0.44, 1.02)	0.06
**Change in type of feeding**												
**Infant formula only**				61/162	1(ref.)	.	92/296	1(ref.)	.	107/383	1(ref.)	.
**Previously breastfed, currently infant formula only**				**35/134**	**0.58 (0.35, 0.98)**	**0.04**	26/87	0.95 (0.55, 1.64)	0.85	18/94	0.67 (0.37, 1.22)	0.19
**Previously exclusive breastfeeding or any breastfeeding, currently any breastfeeding**				**44/179**	**0.54 (0.33, 0.89)**	**0.01**	40/162	0.76 (0.48, 1.20)	0.24	36/181	0.68 (0.43, 1.07)	0.10
**Exclusive breastfeeding since birth**				**105/418**	**0.58 (0.38, 0.88)**	**0.01**	73/348	0.61 (0.41, 0.90)	**0.01**	**42/234**	**0.62 (0.40, 0.95)**	**0.03**
**Duration of breastfeeding**												
**No breastfeeding**				68/181	1(ref.)	.	63/181	1(ref.)	.	57/181	1(ref.)	.
**Stop breastfeeding before the X**^a^ **month**				**28/109**	**0.57 (0.33, 1.00)**	**0.05**	57/216	0.64 (0.41, 1.02)	0.06	77/321	0.69 (0.45, 1.06)	0.09
**Continuing to breastfeed in the X**^**a**^ **month**				**146/576**	**0.55 (0.38, 0.81)**	**0.002**	**108/469**	**0.53 (0.36, 0.80)**	**0.002**	**66/364**	**0.45 (0.29, 0.70)**	**< 0.001**

adjusted for maternal smoking, maternal occupational status, twin pregnancy, gestation length, birth order, daycare attendance and months with which he begins kindergarten

*aOR* Adjusted Odds Ratio

*CI* Confidence Interval

^a^ X is the time month

When we evaluated the change in the type of breastfeeding at 2 months after birth, we observed that not only exclusive breastfeeding but also those who had been mixed feeding was associated with 42–46% lower bronchiolitis risk compared to those who were exclusively fed with infant formula in those 2 months (aOR 0.58, 95% CI 0.35, 0.98 for infant formula at 2 months and previously breastfeeding or mixed feeding and aOR 0.54, 95% CI 033, 0.89 for mixed feeding at 2 months and previously breastfeeding or mixed feeding).

**Fig. 3:**
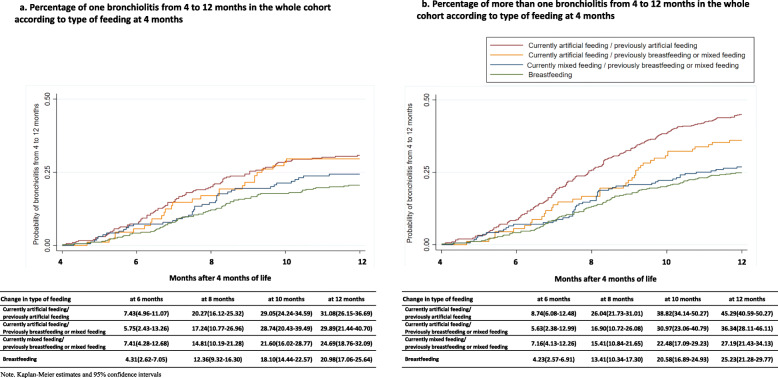
Probability of bronchiolitis (**a**) and number of episodes of bronchiolitis (**b**) from 4 to 12 months in the whole cohort according to duration of breastfeeding at 4 months

Figure 3a illustrates the probability of bronchiolitis according to change in the type of feeding in the first 4 months of life. Children artificially fed at 4 months were more likely to have bronchiolitis than those exclusively or mixed breastfed at 4 months.

Regarding duration of breastfeeding, we observe that the longer the breastfeeding duration, the stronger the protection against bronchiolitis. For instance, maintaining breastfeeding for 2 months was associated with 45% lower risk of bronchiolitis compared to infant formula only (aOR 0.55; 95% CI 0.38, 0.81). While breastfeeding for more than 6 months reduced the risk of bronchiolitis by 55% between 6 and 12 months of life compared to infant formula (aOR 0.45; 95% CI 0.29, 0.70).

**Fig. 4:**
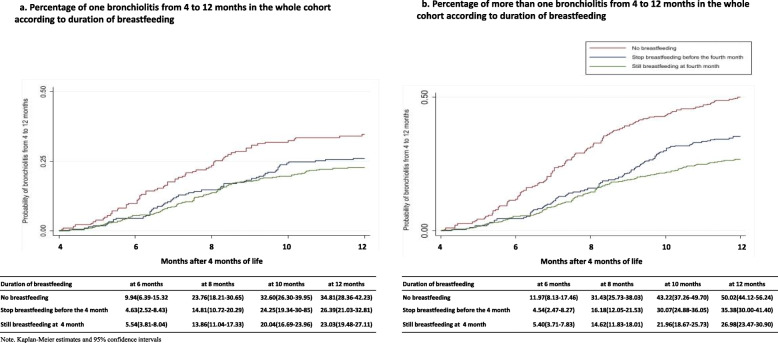
Probability of bronchiolitis (**a**) and number of episodes of bronchiolitis (**b**) from 4 to 12 months in the whole cohort according to type of feeding at 4 months

Figure 4a shows the probability of bronchiolitis according to duration of breastfeeding up to 4 months. Children who never breastfed had more bronchiolitis than those who were breastfeeding at least 4 months (24% vs. 14% at 8 months, and 35% vs. 23% at 12 months).

### Breastfeeding and number of episodes of bronchiolitis

Table 3 shows the relationship between the type of breastfeeding and the number of episodes of bronchiolitis. Results are presented as adjust Incidence Ratios (aIR).

**Table 3:** Relationship between type of feeding and number of episodes of bronchiolitis

	Hospital discharge to 12th month				2nd to 12th month		4th to 12th month			6th to 12th month		
Category	no. bronchiolitis / children at discharge	aIR (95% CI)	p	no. bronchiolitis / children at 2nd month	aIR (95% CI)	p	no. bronchiolitis / children at 4th month	aIR (95% CI)	p	no. bronchiolitis / children at 6th month	aIR (95% CI)	p
**Type of feeding**												
**Infant formula only**	120/163	1(ref.)	.	190/295	1(ref.)	.	217/383	1(ref.)	.	207/477	1(ref.)	.
**Mixed (breastfeeding and infant formula)**	**115/264**	**0.64 (0.49, 0.84)**	**0.001**	**63/179**	**0.59 (0.44, 0.79)**	**0.000**	**53/162**	**0.63 (0.47, 0.86)**	**0.004**	**44/181**	**0.63 (0.45, 0.87)**	**0.006**
**Breastfeeding**	**179/479**	**0.59 (0.46, 0.75)**	**0.000**	**142/418**	**0.62 (0.49, 0.78)**	**0.000**	**101/348**	**0.59 (0.46, 0.76)**	**0.000**	**61/234**	**0.69 (0.51, 0.93)**	**0.016**
**Change in type of feeding**												
**Infant formula only**				115/161	1(ref.)	.	178/296	1(ref.)	.	185/383	1(ref.)	.
**Previously breastfed, currently infant formula only**				75/134	0.79 (0.58, 1.06)	**0.114**	39/87	0.83 (0.58, 1.19)	0.298	**22/94**	**0.56 (0.36, 0.88)**	**0.012**
**Previously exclusive breastfeeding or any breastfeeding, currently any breastfeeding**				**63/179**	**0.53 (0.39, 0.73)**	**0.000**	**53/162**	**0.61 (0.45, 0.83)**	**0.002**	**44/181**	**0.57 (0.41, 0.80)**	**0.001**
**Exclusive breastfeeding since birth**				**142/418**	**0.56 (0.43, 0.73)**	**0.000**	**101/348**	**0.57 (0.44, 0.74)**	**0.000**	**61/234**	**0.63 (0.46, 0.85)**	**0.003**
**Duration of breastfeeding**												
**No breastfeeding**				139/181	1(ref.)	.	125/181	1(ref.)	.	102/181	1(ref.)	.
**Stop breastfeeding before the X**^a^ **month**				**43/109**	**0.48 (0.34, 0.68)**	**0.000**	**94/216**	**0.58 (0.44, 0.76)**	**0.000**	**117/321**	**0.63 (0.48, 0.82)**	**0.001**
**Continuing to breastfeed in the X**^a^ **month**				**213/576**	**0.52 (0.41, 0.65)**	**0.000**	**149/469**	**0.51 (0.40, 0.66)**	**0.000**	**90/364**	**0.50 (0.37, 0.67)**	**0.000**

adjusted for maternal smoking, maternal occupational status, twin pregnancy, gestation length, birth order, daycare attendance and months with which he begins kindergarten

*aIR* Adjusted Incidence Ratio

*CI* Confidence Interval

^a^ X is the time month

Our data show that exclusive breastfeeding and mixed breastfeeding reduce the number of episodes of bronchiolitis when compared with infant formula. Regarding feeding at hospital discharge, exclusive breastfeeding cut down the number of episodes of bronchiolitis by 41% (aIR 0.59, 95% CI 0.46, 0.75) and mixed feeding by 36% (aIR 0.64, 95% CI 0.49, 0.84). Similar reductions in the number of episodes of bronchiolitis were achieved when analysing type of feeding at 2nd, 4th and 6th months.

When we evaluated the change in the type of feeding, we found that an early cessation of breastfeeding before 2nd or 4th months, was associated with a higher incidence of bronchiolitis episodes (aIR 0.79, 95% CI 0.59, 1.06 if introduction of infant formula before 2nd month, and aIR 0.83, 95% CI 0.58, 1.19 if introduction of infant formula before 4th month), while changing from exclusive breastfeeding to mixed feeding had no effect on aIR.

Figure 3b illustrates the probability of and the number of episodes of bronchiolitis according to change in the type of feeding in the first 4 months of life. Children with infant formula at 4 months were more likely to have bronchiolitis than those breastfed at 4 months.

On the other hand, when assessing the duration of exclusive breastfeeding in the first 2 months and the risk of recurrent bronchiolitis between 2 to 12 months of life, we observed that maintaining breastfeeding for 2 months was associated with 48% lower risk having another bronchiolitis episode (HR 0.52; 95% CI 0.41, 0.65). A similar result was observed for those who were breastfed for at least 4 months regarding the risk of suffering one more bronchiolitis between 4 and 12 months. Likewise, maintaining exclusive breastfeeding for 6 months was associated with 50% risk reduction between 6 and 12 months of life (HR 0.50; 95% CI 0.37, 0.67).

Figure 4b shows the probability of and the number of episodes of bronchiolitis according to duration of breastfeeding up to 4 months. In the whole cohort children, who took infant formula had a higher number of episodes of bronchiolitis at 12 months compared to those who breastfed for at least 4 months (50% vs. 27%).

## Discussion

According to our results, any breastfeeding was associated with lower risk of bronchiolitis and lower number of bronchiolitis episodes in this cohort of about one thousand neonates. Early cessation of breastfeeding before the second or fourth month of life was linked to both risk of bronchiolitis and number of bronchiolitis episodes between those babies breastfed and those fed with infant formula. Furthermore, our results showed that the duration of breastfeeding, regardless of the exclusivity of breastfeeding (breastfeeding and mixed breastfeeding) from 0 to 6 months, is a crucial factor in reducing the incidence of bronchiolitis and the number of episodes of bronchiolitis in the first year of life. This may suggest that a longer duration of breastfeeding (regardless of whether it is mixed or exclusive) could reduce the risk of bronchiolitis in the first years of life, and this also suggests that continued exposure to breastfeeding may play a significant role in preventing adverse respiratory outcomes, thus resulting in a lower medical care.

Other studies that have assessed the duration of breastfeeding have also observed a lower risk of adverse respiratory outcomes [23–26]. Lanari et al. also noted that breastfeeding, even in association with formula milk reduced the risk of hospitalization for bronchiolitis during the first year of life [27]. Other studies have also found this association. Thus, Dogaru et al., in a systematic review and meta-analysis, found that the stronger protective effect of breastfeeding was observed in the age group 0 to 2 years for both “asthma ever” and “recent asthma” events regardless of the length or exclusive breastfeeding [28]. Also in line with our findings, the study published by Davisse-Paturet found an association between duration of breastfeeding and its protective effect against bronchiolitis [29]. Our results are also supported by the study by Dekker et al., who found in a prospective cohort of 5675 children, that infant formula was associated with an increased risk of late and persistent wheezing compared to any type of breastfeeding [30]. However, other studies that have studied this relationship have failed to find any association. Thus, Leung et al. did not find an association between exclusive or partial breastfeeding for 3 months or more and hospitalization for asthma, bronchitis and bronchiolitis at age twelve [31]. Nenna et al., in their study of 213 infants hospitalized in Italy, noted that breastfeeding for more than 3 months was associated with increased risk of bronchiolitis [32]. The authors speculated that their findings could reflect the transmission of respiratory infection from mother to child during breastfeeding [32], although the authors did not discuss the possibility of reverse causality (i.e., mothers continuing breastfeeding because they were concerned about the vulnerability of their babies), which could not be ruled out in their case-control design. Our study, however, was designed with prospective follow-up, which allows us to properly identify that bronchiolitis episodes occurred after the feeding period we were analysing each time.

If the lower risk of bronchiolitis we have found is a true protective effect of breastfeeding observed in the first year of life, it may be due to the fact that breast milk contains substances that may have biological effects that can promote lung growth and improve lung function [33, 34]. It has been shown that breastfed children have higher lung volume at the age of 10 years and an investigation attributed this advantage to the mechanical stimulus associated with sucking chest in the early years of life [35]. In addition, the transforming growth factor (TGF) -β contained in breast milk is inversely associated with the risk of having wheezing episodes during the first year of life [36]. On the other hand, breast milk contains numerous anti-inflammatory and immunological agents [37]. This makes breastfed infants perform superior functions than formula-fed infants [38]. In addition, breastfed infants have fewer respiratory tract infections in the first years of life [36] and infections of the lower respiratory tract is the main established risk factor for bronchiolitis. In this regard, Li et al. found that the mothers of infants with bronchiolitis had lower IgG concentration in breast milk. IgG from breast milk could be absorbed by infants, which could play an important role in resistance to bronchiolitis [39].

The main strength of our study is that a homogeneous prospective follow-up of women and children has been carried out in a single centre committed to breastfeeding practices with a relatively large number of participants. In addition, the children’s medical records have been reviewed to objectively know the presence of bronchiolitis and the date of the bronchiolitis, thus avoiding the parents’ recall bias.

However, our study also has some limitations. First, although the size is close to 1000 children, some categories of the analysis have few participants, for example, breastfeeding from the 6th month, this means that we could not study the effect of continued breastfeeding. Harvey et al. in a recent study, observed that breastfeeding for more than 6 months versus “never” was associated with decreased risk of infant wheezing [23]. Second, as breastfeeding information is self-reported, we could have made a reporting bias, since women could have informed in accordance with social expectations and not according to their actual practice.

## Conclusions

The type of breastfeeding used in the first 6 months of life and the duration of breastfeeding impacts the probability of having one, or more than one bronchiolitis in the first year of life. Breastfeeding and mixed feeding, compared to infant formula only, protects against the development of bronchiolitis and the number of episodes of bronchiolitis in the first year of life. Therefore, promoting exclusive or mixed breastfeeding is an easy to implement and economical measure that would be effective in preventing bronchiolitis in infancy.

## Data Availability

Data cannot be made publicly available in order to protect infant privacy. The data is available on request from the University of Cantabria Archive (http://repositorio.unican.es/) for researchers who meet the criteria for access to confidential data. Requests may be sent to the Ethics Committee (ceicc@idival.org) or Dr. Carolina Lechosa-Muñiz carolina.lechosa@scsalud.es).

## Abbreviations

aIR: Adjusted Incidence Ratio
aOR: Adjusted Odds Ratio
CI: Confidence Interval
HUMV: Hospital Universitario Marqués de Valdecilla
SD: Standard deviation
